# Retraction: Tidal Stretches Differently Regulate the Contractile and Cytoskeletal Elements in Intact Airways

**DOI:** 10.1371/journal.pone.0299553

**Published:** 2024-02-22

**Authors:** The PLOS ONE Editors

After publication of this article [1], concerns were raised about similarities between some GAPDH loading control bands (hereafter, LCs) in western blots in Figs 2, 3, and 7.

Specifically,

- The LCs in Fig 2B, Fig 3B (when flipped horizontally), and the lefthand panel of Fig 7B appear similar.
- The LC panels in Figs 2A, 2C, 3A, 7C, and the lefthand panel of 7D appear to show the same data; of note, Fig 7D represents different experimental conditions than the other panels listed here (mechanical loading with versus without constriction). Specific bands are labeled as representing different experimental groups across these figures, and for some panels the bands appear in different order and/or orientation. In the case of Fig 3A, the same band appears to represent both the T0 and D conditions.
- The LCs for Fig 3C appear similar to the LCs in Fig 7A.
- The LCs appear similar between the lefthand panel of Fig 7A and the righthand panel of Fig 7A.
- The LCs appear similar between Fig 2D and Fig 3D.

During editorial follow up, the corresponding author provided the available underlying blot images for these published figures, as well as replicate blot data. Additional methodological information regarding the western blot experiments was also provided to clarify that some loading control panels (left- and right-hand panels of Fig 7A; Fig 2D and Fig 3D) were correctly duplicated because the same membrane was re-probed for different proteins of interest. However, some original blot data needed to address the concerns are now unavailable and so questions about the reliability of the published loading control blots could not be fully resolved. The corresponding author requested retraction of this article.

In light of the unresolved concerns about the reliability of loading control image data in the published figures, the *PLOS ONE* Editors retract this article.

EBS and BS agreed with retraction and stand by the article’s findings. ASLP, BCH, and KRL either did not respond directly or could not be reached.
